# Suicide postvention in psychiatry

**DOI:** 10.1192/j.eurpsy.2021.2196

**Published:** 2021-08-13

**Authors:** L. Castro, C. Desport, J. Freitas

**Affiliations:** 1 Psychiatry, Hospital Magalhaes Lemos EPE, Porto, Portugal; 2 Psychiatry, Hospital Magalhães Lemos, Porto, Portugal

**Keywords:** Suicide postvention, psychiatry

## Abstract

**Introduction:**

During their career and sometimes during their training psychiatrists have to deal with the suicide of patients and have also to help other colleagues and families to cope with the situation. Suicide postvention should be integrated in psychiatric training and better implemented in both psychiatric and community contexts.

**Objectives:**

To discuss the concept of suicide postvention in Psychiatry. To discuss the importance of addressing suicide postvention in psychiatric settings including during specialized training of Psychiatry residents.

**Methods:**

MEDLINE and PubMed databases searches for peer-reviewed studies, published in the last ten years, using combinations of the key-words suicide postvention and psychiatry. Selection of the relevant studies according to the study aims.

**Results:**

The literature search retrieved a total of 44 papers. All the articles that didn´t refer to the studied topic were excluded. Relevant articles discussing suicide postvention were selected, comprising a total of 22 articles. The reviewed papers discuss different contexts and strategies of suicide postvention that will be explored and critically discussed.

**Conclusions:**

Addressing the topic of suicide postvention and implementing support programs and training in this field is crucial to psychiatry trainees, psychiatrists and other mental health professionals that integrate multidisciplinary teams, patients and their families and also to the community as a whole. Future research in this field can help to improve suicide postvention across different settings.

**Disclosure:**

No significant relationships.

